# Building partnerships for linking biomedical science with traditional knowledge of customary medicines: a case study with two Australian Indigenous communities

**DOI:** 10.1186/s13002-019-0348-6

**Published:** 2019-12-23

**Authors:** Joanne Packer, Gerry Turpin, Emilie Ens, Beatrice Venkataya, Mbabaram Community, Yirralka Rangers, Jennifer Hunter

**Affiliations:** 1 NICM Health Research Institute, Western Sydney University, Locked Bag 1797, Penrith, NSW 2751 Australia; 2 Queensland Herbarium, Department of Environment and Science, Mount Coot-tha Botanical Gardens, Mount Cooth-tha Road, Toowong, QLD 4066 Australia; 3 Tropical Indigenous Ethnobotany Centre, Australian Tropical Herbarium, James Cook University, McGregor Road, Smithfield, QLD 4879 Australia; 4 Department of Environmental Sciences, Faculty of Science and Engineering, Macquarie University, Sydney, NSW 2109 Australia; 5 Watsonville Aboriginal Corporation, 141 Atherton Road, Malanda, QLD 4885 Australia; 6 Laynhapuy Homelands Aboriginal Corporation, PO Box 1195, Nhulunbuy, NT 0881 Australia; 7 Menzies Centre for Health Policy, Sydney School of Public Health, Faculty of Medicine and Health, The University of Sydney, Camperdown, NSW 2050 Australia

**Keywords:** Traditional medicine, Community engagement, Collaboration, Bioactivity, Ethnomedicine, Aboriginal, Indigenous

## Abstract

**Background:**

Customary medicine of Australia’s Indigenous peoples draws upon knowledge developed through millennia of interaction with Australia’s unique flora and fauna. Many Indigenous Australians are interested in developing modern medicinal and commercial translations of traditional knowledge; however, barriers of trust and benefit sharing often thwart progress.

**Methods:**

Using a participatory action research framework, university researchers collaborated with members of two Australian Indigenous communities to investigate selected medicinal plants and locally made bush products. A trusted community liaison facilitated the collaboration that was fostered through bilateral site visits. Material transfer and confidentiality agreements ensured that the plant materials were only used for the agreed purpose. Plain language written reports of the laboratory results were provided to the communities with follow up discussions.

**Results:**

In case study 1, only some of the traditional uses for the raw plants were shared with the researchers. Deidentified plants were assessed for antioxidant and antimicrobial properties. In case study 2, the plant names, traditional uses, and preparation methods were shared with the aim of learning more about their plants, potential uses, and optimising their bush products. Literature reviews were conducted that also helped guide in vitro testing of the crude and solvent partitioned extracts. These differences reflected the community’s reasons for conducting the research and intellectual property considerations. In both cases, observed benefits included building trust and strengthening working relationships for ongoing collaboration, fostering enthusiasm for linking traditional and scientific knowledge, promoting cross-cultural learning about scientific methods and traditional medicine, maintaining the relevance of traditional knowledge in the modern world, and initiating community discussions around their bush medicine product development.

**Conclusions:**

Community-driven scientific investigation of traditional medicinal knowledge can facilitate culturally meaningful outcomes, with potentially wide-reaching direct and indirect benefits. Community liaisons were invaluable for establishment of strong relationships and ensured that the research was culturally and locally appropriate. The need for clearer guidelines and regulation around community-driven biomedical research of their plants was identified. Australia would benefit from a user-friendly, open-source toolkit that promotes use of local traditional medicines, contains information about processes and protocols that communities and scientists could use to develop collaborative projects, and guides regulation and ethical commercialisation. Close consultation and collaboration with communities and researchers will be needed to ensure that such a toolkit is culturally appropriate and fit-for-purpose.

## Background

### Intersections of traditional and modern medicine

In many places across the world, traditional medicine (TM) plays an integral or complementary role in local healthcare as widely reported in the *Journal of Ethnobiology and Ethnomedicine*. The World Health Organization, in its Traditional Medicine Strategy (2014–2023), promotes the integration of TM into contemporary healthcare practices and policies commenting “TM, of proven quality, safety, and efficacy, contributes to the goal of ensuring that all people have access to care” [1]. While there have been efforts to integrate Indigenous and traditional medicinal systems into modern medicine and healthcare globally [2–5], there remains a lack of centralised response to this commitment in Australia [6, 7]. This is partly due to the serious ethical concerns around intellectual property, bioprospecting, and benefit sharing that, in Australia, as elsewhere in the world, have led to mistrust and lack of cooperation between traditional knowledge holders and biomedical scientists [8–11]. This paper seeks to contribute to discussion about ways to establish ethical cooperation between traditional knowledge holders and biomedical sciences, not only for increased pharmaceutical sources but also for improved Indigenous community health and empowerment. As such, we describe two case studies that used participatory action research to explore local preferences and requirements for conceptual and methodological collaborations in ethnopharmacology [12].

### Australian Indigenous medicine

Aboriginal and Torres Strait Islander peoples are the Indigenous people of Australia who have been developing and utilising medicinal systems using native flora, fauna, and abiotic materials (e.g. soil) for over 65,000 years [13] and are still done in many places [11, 14, 15]. This knowledge is not static and continues to evolve; as such, the term customary medicine [16–18] is used throughout this manuscript to refer both traditional and contempory Indigenous medicinal practices. Locher et al. (2013) state that the use of plants as therapeutic agents and as interventions for general wellbeing has arguably contributed to their longstanding survival [19]. Traditional knowledge is maintained through intergenerational oral knowledge transmission, and more recently, has been documented to prevent loss and further erosion in post-colonial Australia [20]. Many examples of documented Australian customary medicine exist that have been published both locally and more broadly targeted formats [21–24].

In response to concerns about the increasingly popular bush foods, which in Australia are regulated as foods rather than medicine, in 2001, the Rural Industries Research and Development Corporation released a report on the safety of Australian bush foods [25]. This report compiled existing botanical, chemical, and toxicological information on the current major commercial bushfoods as well as their history of use in Aboriginal culture [26]. Similarly, to enhance broader acceptance and use for customary medicine, improved understanding around preparation methods, chemical, and bioactive properties and interactions with other pharmaceutical and natural medicines would be beneficial.

The importance of scientific evaluation of customary medicine is recognised by communities, health services, and regulators. The Australian Therapeutic Goods Administration (TGA) accepts traditional indications as a valid form of clinical evidence for label claims on listed complementary medicines (AUST-L) and regulates them accordingly as ‘low risk’ therapeutic products. The evidence required by the TGA for a traditional claim is generally based on three generations of documented use [27], which can present a challenge for the recognition of orally transmitted knowledge, such as in an Australian context. In contemporary medical settings, it is advantageous that customary medicine products be scientifically characterised to facilitate an understanding of their biomedical value, efficacy, safety, and interactions with other medicines.

Indigenous people’s health and wellbeing is often associated with cultural connection to their Country [28, 29], an area of land, river, or sea that is the traditional land of each Aboriginal language group or community [30]; however, the evaluation of the biomedical efficacy of customary medicine can also have direct implications on the health of Indigenous people. Indigenous people can be reluctant or experience other impediments to accessing Western health services [31, 32]. Similarly, without scientific evaluation, there can be reluctance by health professionals to acknowledge the potential safety, effectiveness, and value of customary medicine. The promotion of the safe use of customary medicine has the capacity to facilitate a broader understanding and appreciation of Indigenous wellbeing through connection to Country. For Indigenous Peoples of Australia, as stated by Smyth [33], ‘Country refers to more than just a geographical area: it is shorthand for all the values, places, resources, stories and cultural obligations associated with a geographical area’. Indigenous Peoples sing Country, talk about Country, heal Country, visit Country, worry about Country, and long for Country in a way they would talk about a person [34].

There have been grass roots efforts across Australia to preserve and enhance the traditional knowledge of customary medicine through documentation, sometimes driven by the Indigenous communities with whom this knowledge is held [35–37], but often out of curiosity by non-Indigeous people. Locally driven medicinal plants research, with support from medical researchers, can further enhance the potential of customary medicine that may provide direct health and economic benefit to the community. Furthermore, generating scientific data that supports customary medicinal practice can build cultural pride [38, 39], deliver culturally appropriate and accessible healthcare options [40, 41], and provide industry and commercial opportunities for local communities [42]. Inherent differences in ideology and protocols of Western and Indigenous knowledge systems require the development of standards and best practice methods that are built on culturally appropriate experience, transparency, and mutual benefits in order to have a meaningful impact.

This aim of this manuscript is to describe the participatory action research framework used by university researchers, community liaisons, and Australian Indigenous communities to investigate selected customary medicinal plants and locally made bush products; compare the different approaches used, the rationale, advantages and limitation; and identify some of the barriers and facilitators to collaborative laboratory research of customary medicine in Australia.

## Methods

### Methodologies for working with Indigenous communities

For true collaboration to be undertaken, participants agreed on the intent of the co-research, which relevant people/organisations would be involved, and what parts of the project would be done together. Current best practice for work with Indigenous communities necessitates the full and active involvement of Indigenous people at all stages of the research process [43–45]. When led by the community, such collaborations ensure any developments or outcomes accurately reflect the needs and cultural sensitivities of the community and empower communities to be proactive in the adoption of potentially new approaches. The Australian National Health and Medical Research Council and the Australian Institute of Aboriginal and Torres Strait Islander Studies [45] have guidelines for engaging in research with Aboriginal and Torres Strait Islander people to ensure these collaborations are strong and respect the rights and desires of the participants.

An avenue for best ethical practice in research programs with Indigenous people is using a Participatory Action Research (PAR) framework [43–46]. Using PAR, the aim is to provide a democratic basis of collaboration by encouraging both the researchers and community members to work together through an evolving and dynamic process to most effectively collect the information required to affect change. This involves self-reflection (by both parties) throughout the research process to maximise the effectiveness and acceptance of the methods being implemented and thus the co-creation of knowledge.

Time is a required resource to initiate strong working relationships between Indigenous community groups and academic institutions; however, time is often inhibitory with timeframes determined by the funding and administrative bodies. Expedient relationship and trust building between communities and researchers can discourage full and proper engagement and understanding between the parties. Full appreciation of the cultural structures and protocols inherent in both settings is required in order to develop considered protocols and agreements to perform truly collaborative and equitable research. To this end, the involvement of a liaison with cultural knowledge [47] and experience of working in both an academic and community setting can prove invaluable to the success of the collaboration. In both case studies presented here, the research was facilitated and negotiated by a trusted community liaison: in the Mbarbaram case study, by a member of the community itself who was a trained ethnobotanist and government employee; and in the Yirralka case study, an ecologist who had worked with the Ranger group for over 10 years. The university research team also had prior professional interactions with these liaisons which enabled a strong three-way partnership that was instrumental for the research.

### Jointly establishing research protocols

In line with the United Nations Declaration on the Rights of Indigenous Peoples (Article 31(1)), the maintenance, control, protection, and development of traditional knowledge and cultural expression should remain with the Indigenous peoples from whom this knowledge has come [48]. Accordingly, this should be reflected in any research agreements and protocols covering the research collaboration.

Protocols are a set of conventional principles and expectations for consent procedures, attribution, and integrity that are designed to protect Aboriginal and Torres Strait Islander cultural and intellectual property rights. Golvan and Janke (2007) state that ‘Indigenous intellectual property is an integral part of Indigenous Heritage’. Securing Indigenous culture and intellectual property ensures the community (1) provides informed consent, (2) is recognised as the primary guardians and interpreters, (3) has authority to approve or refuse use, (4) benefits commercially from authorised use, (5) can prevent derogatory use, and (6) has the right to maintain secrecy [49].

### Working together: case studies from the NICM Health Research Institute

The two case studies detail collaborations of NICM Health Research Institute (NICM) at Western Sydney University with two Aboriginal Australian community groups: the Mbabaram community of Far North Queensland, and the Yirralka Rangers (YR) of North East Arnhem Land, Northern Territory.

The Mbabaram peoples’ traditional lands extend from the Walsh River just north of Dimbulah (northern Queensland) to the Mt Garnet region, and from west of Herberton to Almaden. It straddles the Great Dividing Range at the headwaters of the Walsh river and includes the townships and settlements of Emuford, Watsonville, Dimbulah, Mutchilba, Leafgold, Petford, Irvinebank, Lappa Junction, and Almaden.

Most Mbabaram people, like many other Australian Aboriginal communities, have experienced cultural impacts since colonisation and do not regularly speak their traditional language and are not entirely familiar with their traditional knowledge (including plant knowledge) that is integral to their culture and wellbeing on Country. Only 300 words from the Mbabaram language are documented, with only a limited number used to identify plants. Few Elders and knowledge holders are custodians of orally transmitted language and plant knowledge.

In response to aspirations of Cape York Indigenous community members to preserve local Aboriginal plant knowledge, TIEC was established in 2010 as a partnership between the Australian Tropical Herbarium, Traditional Owners, the Queensland Government, the Commonwealth Scientific and Industrial Research Organisation (CSIRO) and the Cairns Institute at James Cook University. Based in the Australian Tropical Herbarium, TIEC is the first Indigenous-led government funded Ethnobotany Centre in Australia [50]. As an Indigenous-driven initiative, TIEC aims to engage, support, and build capacity of Traditional Owner groups in tropical north Queensland. TIEC records and uses Indigenous ethnobiological and ethnoecological knowledge for cultural use on Country [51].

The Yirralka Rangers (YR) are a business unit of the Laynhapuy Homelands Aboriginal Corporation (LHAC), an Aboriginal owned and managed community organisation based in Yirrkala in north east Arnhem Land in the Northern Territory, Australia. Established in 1985 in the wake of the Homelands Movement of the 1970s, LHAC has grown to be the major service provider to over 1,000 Yolngu living in 30 homelands across the region. LHAC also operates through two other lines of business (Health and Homeland Services) and additionally provides Aged Care and Youth programs.

The YR formed in 2003 in response to the desire of Traditional Owners to protect the cultural and environmental values of their land and control its management. They have developed into a significant area of employment for Yolngu living on homelands and provide career paths which enable Yolngu to maintain their connections to Country while participating in the Australian economy. The YR carry out a range of land and sea activities within the Laynhapuy Indigenous Protected Area including feral animal and weed management, biodiversity monitoring, marine management, cultural heritage protection, fire management, visitor management, and community engagement activities. Taking advantage of their Indigenous ecological knowledge, the Yirralka Miyalk (Women’s) Rangers additionally collect and grow native plants for their homeland communities and have also developed a successful body products business using natural ingredients found in Yolngu Country.

Yolngu people retain strong links with their customary medicines with a number of pharmacopoeias of local bush resources have been published [52–54]. They also have a keen and longstanding interest in the ‘both ways’ approach which draws on Indigenous and Western knowledge systems [55].

The YR commenced their bush product enterprise in 2011 with the assistance of an external organisation, the Aboriginal Bush Traders. The enterprise has since evolved and involves the YR collecting local bush medicine plants and preparing the plants to make body scrubs, soaps, and rubs which they sell to local retail outlets and at markets and festivals in ‘The Top End’.

NICM worked with the two community liaisons and their respective Aboriginal communities to build capacity and an understanding of the potential medicinal value of selected customary medicine. By gaining access to scientific research personnel and laboratory facilities capable of investigating properties of the plant extracts, the communities were able to use the biomedical analyses to increase their understanding and further develop and promote their medicinal resources.

A PAR framework that built on previous collaborative relationships and aligned with current best practice for work with Indigenous communities was used [43–46]. The researchers at NICM were approached by a trusted community liaison for assistance with the investigation of biomedical properties of the communities’ plant material. Working relationships were reinforced through site visits by community representatives to the NICM laboratories, in Sydney, NSW, and by NICM researchers visiting the respective communities.

In both cases, material transfer and confidentiality agreements were established between Western Sydney University and the Aboriginal communities to formalise agreements around the handling and use of plant material and results. The advantages and disadvantages of community sharing options when disclosing information about their plants (e.g. the plant species or common names, traditional uses, or preparation methods) were discussed (Table 1). The level of information agreed to reflect how comfortable the community was with sharing information and their reasons for conducting the research. Written agreements stipulated that any intellectual property produced in the projects would remain the property of the respective community. Aligning with ethical research, any publication would only be possible through co-authorship and on approval of the community representatives. Confidential documentation by NICM included field notes, photographs, and laboratory results.

**Table 1:** Implications of communities disclosing information about their customary plants

	No information disclosed	Selective information disclosed			All information disclosed
		Plant name	Traditional uses	Traditional preparation methods	
Community					
Advantages	• The community controls all traditional knowledge about their bush medicine. • Completely blind and unguided testing could provide the community with new, confidential information about the potential uses, medicinal properties, and/or preparation of their plant.	• Information is collated from other reports and published literature that can increase the depth of information provided to the community. • Able to compare the community’s uses with pre-existing scientific research and published information about other communities who also use the same plant. • Fewer tests may be required that can reduce time and cost.	• Laboratory tests for assessing bioactivity can be selected to correspond with the traditional uses of the community, thus providing preliminary scientific evidence about potential medicinal properties of the plant. • Fewer tests may be required that can reduce time and cost.	• The laboratory results will more closely apply to the traditional preparations used by the community. • The community can use the results to further optimise their bush medicine products, for example, potency or shelf-life. • Fewer tests may be required that can reduce time and cost.	• The community can maximise the researcher’s expertise to provide efficient, timely laboratory analysis that most closely corresponds to the community’s preparations methods and uses. • The community can prepare to commercialise their bush medicine products as both traditional and scientific knowledge will be required for the process, including possible regulatory requirements.
Disadvantages	• Laboratory testing is unguided at all stages and can be more time consuming and expensive. • Results may not align with the community’s traditional uses and preparation methods.	• The community must trust that researchers will maintain confidentiality about the results of the laboratory tests. • Laboratory testing and correlating results may not align with the community’s preparation and uses.	• The community must trust that researchers will maintain confidentiality about traditional uses. • If the laboratory testing only aligns to customary uses, novel applications for the bush medicine may not be discovered.	• The community must trust that researchers will maintain confidentiality about traditional preparation methods. • The community may not be able to replicate the optimal preparation methods identified in the laboratory, for example the extraction solvents may not available to the public or very expensive equipment may be required.	• The community must trust the researchers will maintain and protect all confidential information and intellectual property.
Research team					
Advantages		• Researchers can review the scientific literature to help to guide plant handling, laboratory methods, and testing, review results of previous research, and identify characteristics of the plant to inform their laboratory testing.	• Researchers can build on the community’s knowledge to optimise the selection of the bioactivity tests.	• Researchers can build on traditional knowledge to optimise plant preparation and extraction methods for laboratory testing.	• Researchers can build on all the community’s knowledge to optimise every stage of the laboratory testing.
Disadvantages	• Can be time consuming and expensive. • Testing is limited by the expertise of the research team. • The potential novelty of the results cannot be confirmed as plant name is unknown.	• Plant preparation, extraction methods, and selection of bioactivity tests are limited by the expertise of the research team.	• Plant preparation and extraction methods are limited by the expertise of the research team.	• The bioactivity tests selected are limited by the expertise of the research team.	

## Results

### CASE STUDY 1: Mbabaram community, Far North Queensland

This collaboration formed part of a larger project that was led by the Tropical Indigenous Ethnobotany Centre (TIEC) and involved some of the young Mbabaram trainee land managers in ethnobotanical recording, plant identification, and specimen vouchering skills. The goal was to provide new skills, engage the group, reinvigorate discussion, and use traditional knowledge of the region more generally. NICM laboratories were approached to analyse various raw plant material samples of cultural significance to the Mbabaram people of northern Queensland, as identified through this project.

The collaboration between the Mbabaram community, TIEC, and NICM was initiated by TIEC through a community liaison (author GT), who approached NICM to conduct laboratory analysis of culturally significant medicinal plants identified by the community. Two NICM researchers were invited to visit the Mbabaram community in October 2014 for discussions around a possible collaborative research project. The two groups shared information about their backgrounds and how they might collaborate towards a shared vision of strengthening the scientific and cultural knowledge about plants used in CM. NICM researchers explained the capabilities of NICM and how this might assist to address the needs of their current and future projects of TIEC. It was at this initial meeting a transfer of materials agreement was signed, which similar to a service agreement, ensured all results and information disclosed was confidential and owned by the Mbabaram community.

Following ongoing contact and communication, this pilot collaboration was initiated and funded through CSIRO funding to the Australian Tropical Herbarium. Four representatives from the Mbabaram Aboriginal community visited NICM offices and laboratories in August 2015. During this visit, the community representatives met NICM staff, toured the facilities, and were shown many of the methods used for the standard extraction and laboratory analysis of plant material. The representatives also had the opportunity to visit the nearby Australian Botanic Garden Mount Annan Bush Food gardens.

During this visit, Mbabaram representatives brought with them plant material and information relating to 16 plant parts of 8 species for analysis. To initiate the relationship on good faith and aligning with the rights of Indigenous Cultural and Intellectual Property, confidentiality was ensured by disclosing only the plant family name. After consideration of traditional applications and discussions around specific areas of community interest, capabilities of the laboratory, and time and budgetary constraints, nine plant parts were selected for initial investigation.

Guided by the community representatives and based on their traditional use, the selected plant extracts were assessed for their antioxidant activity and the antimicrobial potential of the extracts assessed against *Staphylococcus aureus*, *Pseudomonas aeruginosa*, *Escherichia coli*, and *Candida albicans.* A formal report of the results of analysis was provided to the community including a plain language summary. Following a face to face meeting to discuss the results, the outcome of the analyses was communicated directly with the broader Mbabaram community by the TIEC liaison.

Throughout the project, the TIEC trainee land managers expressed excitement about learning from the traditional knowledge of their Elders, while gaining first-hand experience with the techniques and methods of Western science to further understand the mechanisms by which these plants might work against microbes/disease. The promising results from the analysis of some of the plant samples has initiated renewed engagement with these bush medicines, with the trainees interested in further investigation, involvement, and communication of the project results.

The collaboration has also facilitated broader community interest in the project, including discussion about how positive results could translate into the development of commercial enterprise such as the development of bush medicine products, the cultivation of medicinal plant material and further investigation of other medicines and bush tucker opportunities, and related strategies to support local tourism and cultural pride. This enthusiasm has spread to neighbouring communities who are also interested in investigating their plant material and products.

Further analyses of some of these plants is ongoing through short student projects funded through NICM, with the results remaining confidential and communicated to the community as they become available. These student projects are also covered by the confidentiality agreements initially established between the parties, with the students agreeing to these confidentiality limitations and IP ownership by the community before commencing work.

### CASE STUDY 2: Yirralka Rangers, north east Arnhem Land, Northern Territory

The second case study involved the biomedical analysis and optimisation of three traditional medicinal plants used in the preparation of the Yirralka Rangers’ (YR) range of natural personal care ‘bush products’. This relationship was facilitated by cross-cultural ecologist (author EE) who had worked with the ranger group for 10 years prior to this collaboration on a range of environmental projects and who acted as community liaison for the project.

NICM researchers were approached through the community liaison (co-author EE) as the YR were interested in scientifically evaluating the bioactive potential of their bush products, and the plants used in their production, in an effort to recognise the medicinal and commercial value of these products. The YR are very proud of their bush products and medicinal knowledge, as articulated by one of the YR members when asked why they wanted to scientifically evaluate their products, they perceived value in two-way learning ‘So all the community can see me, doing bush products … doctors from all over will come to visit the clinic, will know about the bush medicines …, for when visitors come and they can see, and they can learn from us, and we can share our knowledge, and then they can get their medicine from us’.

The YR were interviewed by author BV about their involvement, desires, and traditional knowledge relating to the project, for which appropriate human research ethics clearance was sought. As with the first case study and aligning with best practice, this collaborative pilot project with the YR was also covered by confidentiality agreements between the parties.

This project was funded through a Western Sydney University grant which allowed for reciprocal visits between the researchers from NICM and the community, some consumables and research assistance for the initial analysis of the extracts.

Two NICM researchers (JP, BV), accompanied by EE, visited Yirrkala in October 2015. Discussions were held with community representatives and senior knowledge custodians regarding the traditional knowledge used in the development of the bush products and their interest in two-way research. The NICM researchers were taken out ‘on Country’ by the YR and taught about the medicinal and cultural significance of the plants to be studied. They also visited the local production facility to learn more about the current preparation methods of the bush products. General discussions also included the challenges faced by the community regarding topical infections and the desire to identify and enhance the antimicrobial properties of the topical bush products.

In the following month, two Yirralka Miyalk Rangers (accompanied by the Miyalk Ranger Facilitator and EE) visited NICM facilities in Sydney to meet with the researchers and broader members of the NICM team. The purpose of this visit was to explain and demonstrate the laboratory based testing methods and to further discuss how the potentially optimised products could have applications for local public health. The visiting YR were given the opportunity to gain hands-on experience in the laboratory analysis of their plant material to encourage a thorough understanding of the scientific methods.

Unlike the previous case, the YR provided NICM with the scientific names of the plants, which allowed NICM researchers to review available literature regarding the chemical constituents and bioactivity. Based on these reviews, NICM performed preliminary chemical analysis (RP-HPLC) of the crude and solvent partitioned extracts of each plant part used in the bush products, as well as preliminary bioactivity (antimicrobial and antioxidant) analyses. The results were presented to the community as a report (including a plain language summary) and teleconference discussion explaining the results and the implications for potential further directions of the project.

Following the pilot project, this collaboration continued through the engagement of a Masters of Research student (BV, initially employed as a research assistant on the pilot project). BV subsequently visited the community to present the results of the pilot data to the YR and Laynhapuy Homelands Aboriginal Corporation CEO and confirm the next directions of the project. Guided by the interests of the YR and pilot data, BV’s thesis investigated the antimicrobial and anti-inflammatory activity of the bush medicines, as well as the stability of the plant material when extracted under varying conditions. In comparison the first case study presented, YR identified plant species to researchers, thereby allowing for a comprehensive systematic review to be conducted and thus providing further reporting of the medicinal properties of the medicinal plants studied.

## Discussion

In this paper, we used two case studies to elucidate similarities and differences experienced when establishing collaborative ethnopharmacology research projects by Indigenous groups and biomedical scientists in Australia. Although efforts have been made to identify minimal conceptual and methodological considerations in ethnopharmacology research [10, 12], there is a lack of descriptive local Australian examples for mechanisms of collaboration that seek to derive mutual benefit and maintain high ethical standards. As demonstrated through these two case studies, community-driven research that links biomedical science with traditional knowledge can provide a tool supporting communities seeking to reinvigorate traditional knowledge of customary medicine, develop their commercial products and promote cultural pride. Providing empirical evidence of the biomedical potential of plants of cultural and medical significance can assist economic growth initiatives through commercial product development, horticulture and tourism opportunities, and healthcare practices based on local wisdom.

Similar to other projects [56], the utilisation of a trusted liaison was invaluable and strengthened these collaborations, acting as an intermediary between the NICM research team and the community. The research collaboration was developed via a community liaison who had worked closely with the community for some time on various initiatives and who had also independently established a working relationship with the NICM researchers through previous projects. While the community members were directly involved with devising the project and its development and met regularly with the NICM researchers throughout the project, the liaison was in a position to fully appreciate both the community dynamics and the university research culture. Where necessary, the liaison was able to advocate on behalf of the community and facilitated a culturally sensitive partnership allowing the community-driven projects to proceed in a cost effective and timely manner.

Both projects took measures to ensure protection of intellectual property of the knowledge holding groups, although there were differences in the approaches based on the stage of commercial product development. Of the plants provided for testing by TIEC, NICM researchers were only given the botanical names to the family level. The selection of laboratory tests was informed through the information provided by Mbabaram members about the local traditional medicinal uses of each plant. Consequently, no preliminary research (e.g. scoping literature reviews) could be undertaken, and the results obtained from preliminary testing could not be compared with those of other scientific research or recorded traditional knowledge.

In comparison, the plants provided by the YR, as used in their bush product range, were in the public domain, with both local and scientific names printed on their product packaging and provided on accompanying advertising materials. In addition to testing for the bioactivity of the plants used in their bush products, the YR sought advice about product optimisation. Botanical identification of the plants, detail of the plant parts used in the preparation, and the extraction methods, allowed NICM researchers to conduct literature searches and build on existing scientific knowledge to further support the YR.

In addition to developing protocols with the community, it is necessary to satisfy local council, state, or national requirements, such as permits to conduct scientific research of (and with) Indigenous communities. This has the potential to pose significant challenges for Indigenous communities and scientists alike, who often are not aware of the various permits and processes, which can hinder the timely and appropriate completion of funded research projects. Further guidance is needed at local, state, and national levels to develop consistent, clear processes able to support the growth and momentum of Indigenous community-driven research projects.

Notwithstanding the challenges outlined, the potential for mutual benefits from cross-cultural approaches to PAR are substantial and transformative [29]. Both projects had a significant, positive, and empowering impact on the participants and their broader communities. Future capacity building opportunities could include training local people from across institutions (such as the clinic, school, and ranger programme) to document ethnobiological and ethnoecological knowledge for cultural use ‘on Country’, or establishing infrastructure for the production of their bush medicines. The basis for further collaboration with the scientists at NICM has now been established and the two communities are inspired to engage in further research and data collection.

The Indigenous communities, however, were not the only people to directly benefit from the two projects. The NICM research team had the privilege of learning from their Indigenous colleagues and were given the opportunity to participate in interesting and meaningful research. Sharing knowledge and reciprocal two-way learning was inspirational and laid the foundations for ongoing commitment by NICM to further develop these collaborations and actively support community-driven research.

## Conclusion

Indigenous community-driven ethnobotanical research is ideal for ensuring the ongoing use and preservation of traditional knowledge and intellectual property. Democratic frameworks such as through participatory action research are appropriate methodologies to guide collaboration between researchers and community members. A community liaison who can represent the community as well as navigate the research landscape is invaluable to cross-cultural collaborative research.

By identifying the biomedical value of CM plants and optimising bush products, communities can build on their traditional knowledge to develop and incorporate these medicines into local healthcare practices and share their medicines with the wider Australian community. Other potential outcomes include opportunities for economic development of bush product enterprises and empowering the local community through job growth and cultural pride.

Further development of “how-to” resources is however required for both Indigenous communities and interested researchers to aid the development of local Indigenous medicines and support incorporation into public health initiatives. To improve accessibility to what currently is expert knowledge held by a few individual researchers and teams in Australia, the toolkit should be user-friendly and open-source and include information for academics and lay-people, including those with no science background. The toolkit needs to outline the series of processes required for such research and collaboration including the following: the scientific analysis of plants, products and formulations; guidance around processes and regulations; through to capacity building opportunities within communities. Australia as a whole would benefit from such a resource outlining the requirements of Australian regulatory and governing bodies for the recognition, use, and ethical commercialisation of Australian CMs. A toolkit such as this could be used by communities and scientists to support meaningful collaborative projects and give all members of the partnership access to trusted advice, expertise, and infrastructure to build capacity in this area.

## Data Availability

Data sharing is not applicable to this article as no datasets were generated or analysed during the current study.
